# Material property analytical relations for the case of an AFM probe tapping a viscoelastic surface containing multiple characteristic times

**DOI:** 10.3762/bjnano.8.223

**Published:** 2017-10-26

**Authors:** Enrique A López-Guerra, Santiago D Solares

**Affiliations:** 1 Department of Mechanical and Aerospace Engineering, The George Washington University, Washington, DC 20052, USA

**Keywords:** atomic force microscopy, harmonic functions, tapping-mode AFM, viscoelasticity

## Abstract

We explore the contact problem of a flat-end indenter penetrating intermittently a generalized viscoelastic surface, containing multiple characteristic times. This problem is especially relevant for nanoprobing of viscoelastic surfaces with the highly popular tapping-mode AFM imaging technique. By focusing on the material perspective and employing a rigorous rheological approach, we deliver analytical closed-form solutions that provide physical insight into the viscoelastic sources of repulsive forces, tip–sample dissipation and virial of the interaction. We also offer a systematic comparison to the well-established standard harmonic excitation, which is the case relevant for dynamic mechanical analysis (DMA) and for AFM techniques where tip–sample sinusoidal interaction is permanent. This comparison highlights the substantial complexity added by the intermittent-contact nature of the interaction, which precludes the derivation of straightforward equations as is the case for the well-known harmonic excitations. The derivations offered have been thoroughly validated through numerical simulations. Despite the complexities inherent to the intermittent-contact nature of the technique, the analytical findings highlight the potential feasibility of extracting meaningful viscoelastic properties with this imaging method.

## Introduction

Several current applications demand physical understanding of soft dissipative materials at the nanoscale [1–5]. This type of materials, such as polymers, biological cells and even some metals, has been successfully described with linear viscoelastic theory [6–8] and its characterization at the nanoscale has been performed by various techniques, where the atomic force microscope (AFM) has played a prominent role. Within AFM, quantitative characterization of viscoelastic materials is usually performed through contact-mode methods. Contact-resonance AFM, force-modulation AFM and static force spectroscopy are the most popular examples in this category [9–13]. The permanent-contact nature of these methods offers an important advantage in mechanical characterization. In the case of contact-resonance or force-modulation techniques, where the tip oscillates harmonically in permanent contact with the sample, a steady-state development between force and displacement is achieved, which greatly simplifies the interpretation of the observables. Furthermore its mathematical treatment shares close relationship to the well-established bulk technique, dynamic mechanical analysis (DMA [14–15]). The analytical simplicity afforded by permanent tip–sample contact, however, comes with the shortcomings of loss of accuracy in the acquisition of the topography and sample damage induced by constant tip drag. Additionally, these methods are prone to significant tip wear and contamination which could make quantitative characterization unreliable due to constant changes in tip geometry.

Dynamic methods have been designed to overcome the above issues, whereby tapping-mode AFM is arguably the most popular technique, owing to its versatility, sturdiness and ease of use. This technique can offer accurate topographical measurements, while simultaneously offering material contrast through the phase channel. The acquisition of material contrast through the phase signal, known as phase spectroscopy, has been extensively used, along with the derivation of representative energy quantities that are popularly used for compositional mapping [16–18]. However, the direct correlation of the results with quantitative material properties is not simple.

A number of studies have highlighted the challenges involved in characterizing viscoelastic materials with dynamic intermittent-contact methods [19–22], but further work remains, both in accurately pinpointing the issues involved and in finding robust solutions for them. Typically, viscoelasticity in AFM has been oversimplified in an effort to maintain the analytics tractable, but this has been done at the expense of implementing models that do not properly represent the behavior of real materials, at least with regards to the well-established classical viscoelasticity, which predicts complex time behaviors for samples with multiple characteristic times.

In this work we have adopted a strategy focused on the material point of view, in order to determine what would happen (in terms of force response) if a (properly modeled) viscoelastic material is tapped by a flat indenter probe following an intermittent-contact sinusoidal trajectory. From the rheological viewpoint, the characterization of viscoelastic materials has been classically performed by applying a well-defined input excitation (either stress or sample strain) to elicit a response, which is then measured. The measured output response and the well-defined input are related through a material transfer function, which contains the viscoelastic parameters. Standard inputs are typically strain and stress step functions (in the case of stress relaxation and creep experiments, respectively) or harmonic excitations (in the case of DMA). Following the spirit of classical rheology, we regard tapping-mode AFM as a non-standard excitation of a viscoelastic sample, and exploit the fact that the near sinusoidal nature of the tip deflection in this technique implies that the sample necessarily experiences a portion of that sinusoidal trajectory during the contact period. This strategy has led us to expressions for the tip–sample force in time, in terms of meaningful viscoelastic parameters. Additionally, further manipulation has allowed us to obtain expressions for the energy quantities measurable in a normal tapping-mode AFM experiment, in terms of viscoelastic properties. This connection represents a potentially feasible path towards the meaningful quantitative extraction of viscoelastic properties with tapping-mode AFM.

## Results and Discussion

### Contact problem for viscoelastic surfaces

**Figure 1:**
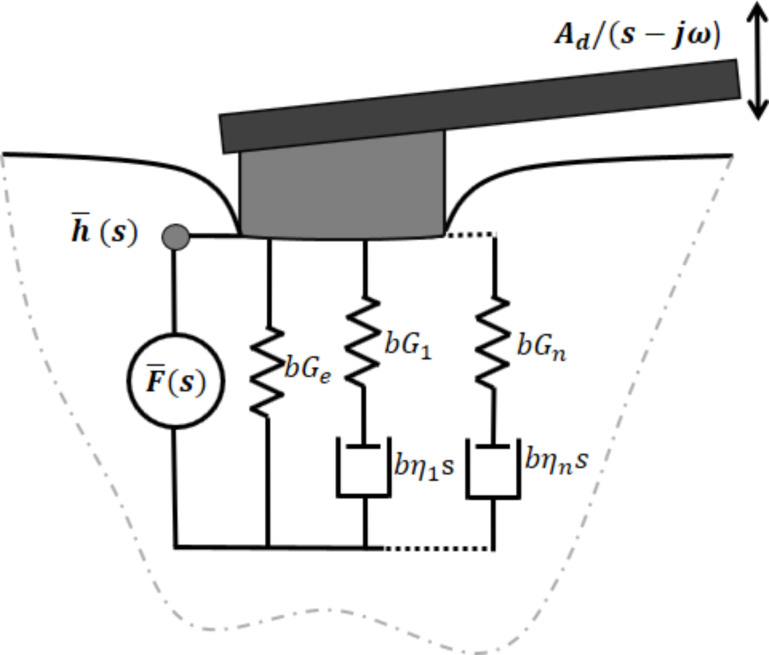
Mechanical model diagram of a flat-end indenter penetrating into a Generalized Maxwell (Wiechert model) viscoelastic surface. The model diagram shows the relationship between the Laplace transformed penetration 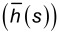 and the transformed force 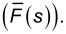 The constant *b* corresponds to a cell constant (with units of displacement) which allows conversion between stress–strain and force–displacement relationships [8]. By applying the correspondence principle to the elastic solution of flat-end punch indentation (derived by Sneddon [29]), it is possible to obtain its viscoelastic counterpart, as previously done by Cheng et al. [28]. For the case of a time-independent Poisson’s ratio, the cell constant reduces to *b* = 4*R*/(1 − ν).

Traditionally, stress analysis for linear viscoelastic materials has been approached using the elastic–viscoelastic correspondence principle (also known as simply “correspondence principle”), a mathematical technique that exploits the fact that the Laplace transformed field equations for linear viscoelastic materials have the same form as the field equations for linear elastic materials [23–24]. The above fact is harnessed to extend the wealth of available elastic solutions to their viscoelastic counterparts [25–27] in the cases where the boundary conditions imposed in the derivation of the elastic solution remain constant in time. For a flat-end indenter penetrating a viscoelastic surface, Cheng et al. have shown that the correspondence principle can be successfully applied because the boundary conditions do not change in time [28], and therefore Sneddon’s elastic solution [29] can be extended to its viscoelastic counterpart. The above leads to a general solution in the Laplace domain in terms of viscoelastic operators, relating the transformed load 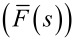 and the transformed displacement 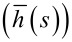 for a flat-end punch penetrating a viscoelastic half-space (with the time-independent Poisson’s ratio, ν) [28]:

[1]
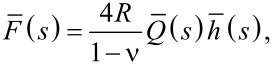


where 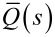 is a ratio of polynomials in the complex variable *s* which is related to the viscoelastic parameters of the sample (further explanation is provided below), and *R* is the circular punch’s radius. The factor 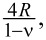 having units of displacement, can be regarded as the cell constant *b* which converts stress–strain to force–displacement relationships [8]. This relationship (Equation 1) is physically represented in Figure 1 for the case of a Generalized Maxwell model with an arbitrary number of characteristic times. The load in Equation 1 may also be written in the time domain as a convolution of the relaxation modulus (*G*(*t*)) with the time derivative of the displacement:

[2]
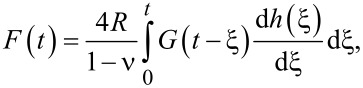


where the substitution 
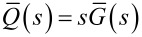
 has been made to express the result in terms of the widely known relaxation modulus, which is the stress response to a unit step strain [8,30] (see Supporting Information File 1 for further details about the relaxation modulus).

### Harmonic excitations in contact mode

Before analyzing the case of intermittent contact between a flat-end indenter and a viscoelastic surface, we turn our attention to the more well-established case of harmonic permanent contact. This case closely coincides with the well-known analytical treatment of dynamic mechanical analysis (DMA) and therefore, we use it as a basis of comparison for our current developments for the intermittent-contact case. Although the case of harmonic excitations in permanent tip–sample contact in AFM is closely related to DMA analysis, a couple of important differences exist.

With the definitions previously introduced, we can now define the force excitation that will be applied in a tip–sample system with permanent-contact harmonic motion, and subsequently derive the associated displacement response. To achieve a steady-state harmonic response in an indenter–sample system, it is required to initially elicit a step force, which translates into a deflection setpoint, as in contact-resonance AFM [10,31] and force-modulation AFM [11]. In addition to that displacement (deflection setpoint), a harmonic excitation is imposed. Mathematically the total tip–sample force excitation is:

[3]
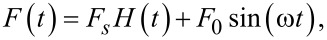


where *F_s_* is the static force setpoint, *H*(*t*) is the Heaviside (unit step) function, *F*_0_ is the amplitude of the harmonic excitation force, ω is the driving and response frequency, and 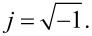 Obviously, one has no direct control over the harmonic tip–sample force in the experiment (similar to contact-resonance AFM or force-modulation AFM), but instead, a displacement or force excitation is imposed on the microcantilever probe, which results in a harmonic displacement at the tip, which in turn leads to a harmonic tip–sample force [11] (second term on the right hand side of Equation 3).

The above excitation force generates a displacement response which can be conveniently derived by using Laplace transforms. The displacement response at steady state is (see Supporting Information File 1 for details on the derivation):

[4]
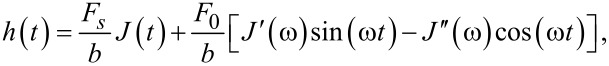


where *J*(*t*) is the creep compliance of the material (strain response to a unit step stress [8,30]), *J*′(ω) is the storage compliance and accompanies the portion of the harmonic response that is in phase with the excitation, and *J*″(ω) refers to the loss compliance, which accompanies the response term that is in quadrature with the excitation. The above can be simplified assuming a long experimental timescale (when a steady-state harmonic response has been developed). Incorporating the fact that lim*_t_*_→∞_ *J*(*t*) = *J_e_* (that is, the compliance of the material approaches the equilibrium compliance, *J_e_*, at long timescales [8]), leads to:

[5]
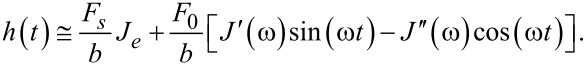


To obtain the energy dissipated per cycle (*E*_cyc_) in the steady state, we integrate the force with the derivative of the position over one cycle:

[6]
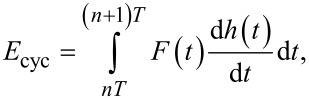


where *T* is the excitation period (*T* = 2π/ω) and *n* is an integer which ensures that the integral in Equation 6 is performed over one period at steady-state. The energy dissipated per cycle is then:

[7]
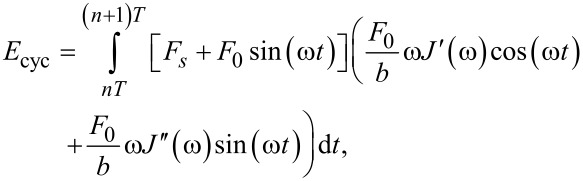


which upon integration yields:

[8]
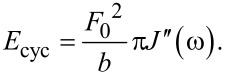


In an analogous way, the virial of the interaction (the elastic energy quantity), defined as the convolution of force with position 
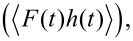
 can be calculated as [18,32]:

[9]
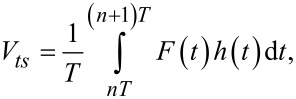


[10]
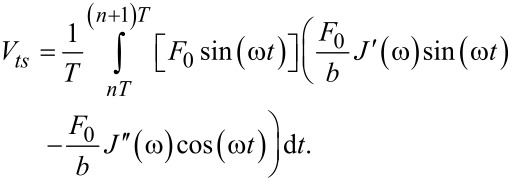


Note that in Equation 10 we have not included the static deflection, keeping consistency with the authors who developed the expression [18,32]. Solving the integrals yields:

[11]
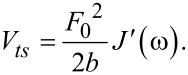


The above coincides with twice the average energy stored per cycle [8]. It also coincides with the maximum coherently storable energy [8,33]. The maximum coherently storable energy (energy stored by the elastic components, corresponding to the in-phase portion of the harmonic response and the equilibrium compliance contribution) occurs at the one-quarter cycle point [33], and is:

[12]
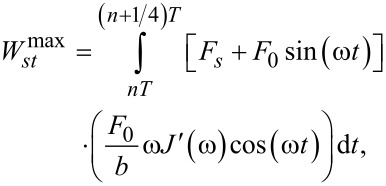


which yields:

[13]
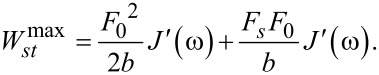


The above is the maximum energy stored per quarter cycle, including the energy stored from the equilibrium modulus due to the static deflection (second term in the right hand side). Thus, in this application when a steady-state response is achieved, the virial of the interaction coincides with the maximum storable energy – if the energy stored by the equilibrium modulus due to the static deflection is not considered. The virial in this case has a very intuitively physical interpretation, which is not the case in tapping-mode AFM, as will be discussed later. Last, the ratio between dissipated energy and virial, in this case, is related to the loss tangent (tan θ(ω)):

[14]
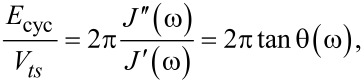


where θ(ω) – the loss angle – describes the phase lag (or lead) of the response of a viscoelastic material to a harmonic excitation in the steady state, and its value spans from zero, when a material is completely elastic, to 90° when the material is completely viscous. By convention, the stress always leads the strain. Note that the above expression is only valid for the case of harmonic excitations when a steady-state response is achieved. As will be shown later, this is not valid for the case of intermittent-contact excitations, where steady-state response cannot be assumed.

### Intermittent contact

**Figure 2:**
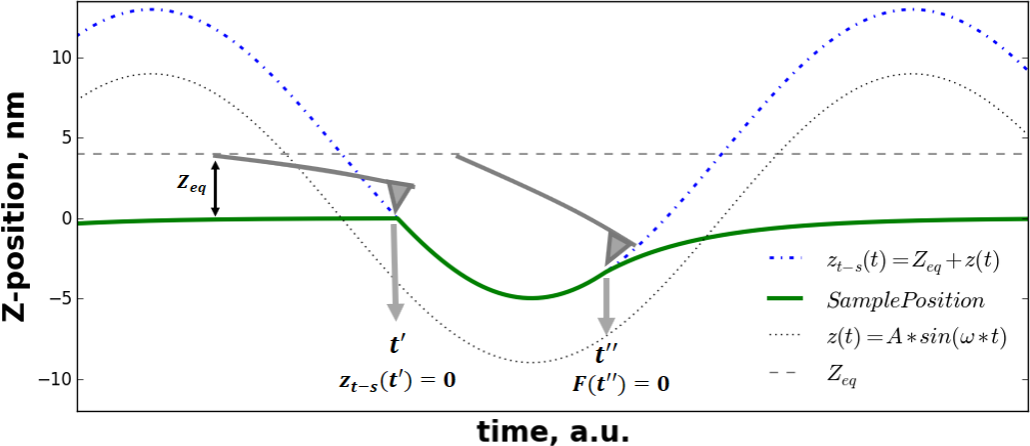
Scheme of intermittent-contact tip–sample interaction in AFM. The figure shows the results of a numerical simulation of a rectangular cantilever with a flat-end tip tapping on a viscoelastic surface. The black dotted line refers to the instantaneous tip deflection. The tip trajectory (blue dash-dotted line) displays a nearly sinusoidal behavior in tapping-mode AFM, typical of high-*Q* environments. The green line corresponds to the sample deformation (instantaneous sample position). The sample viscoelastic parameters have been chosen in the simulation in such a way that it undergoes a relatively large deformation. The above has been done for illustrative purposes. However in typical cases it is expected that the sample deformation will be significantly smaller than tip displacement. The green solid line represents the sample position. From time *t*′ to time *t*″, tip and sample share the same spatial coordinate, which is the key observation for the derivation of the analytical equations in this study. During this time range the deformation can be regarded as the excitation (see Equation 16). Using that displacement excitation, an analytical closed-form relation for the force can be derived (Equation 17). At time *t*′, the tip–sample position is zero (taking as reference the undeformed surface), and from Equation 15 it is clear that: *A*sin(ω*t*′) = −*Z_eq_*. At time *t*″, the tip–sample force becomes zero, which indicates loss of contact between tip and the sample.

Now we turn our attention to the case when a tip interacts in intermittent contact with a viscoelastic half-space. Specifically, we focus on the case of a hard flat-punch indenter penetrating a viscoelastic solid in an intermittent-contact manner, which is a problem relevant to nanoscale spectroscopy techniques, such as tapping-mode AFM [34–35]. In standard tapping-mode AFM, a cantilever is harmonically excited either by imposing an oscillatory motion at the base (acoustic excitation), or an oscillatory force at the tip (magnetic excitation), or by intermittent local heating near the base through a laser. As a result, the tip interacts with the sample intermittently and develops a quasi-steady-state response (Figure 2) [16–17]. The dotted blue line in Figure 2 shows the simulated tip trajectory for an AFM cantilever interacting with a viscoelastic surface in tapping-mode AFM (simulation details are provided in the figure caption). The instantaneous tip–sample distance, taking as reference the undeformed sample surface, is approximately given by:

[15]
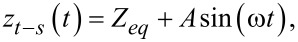


where *Z_eq_* refers to the average tip–sample position, *A* is the tapping amplitude, ω is the excitation frequency, and *A*sin(ω*t*) = *z*(*t*) is the instantaneous tip deflection. We have omitted the phase term, usually expressed as a phase lag between cantilever excitation and response, due to the sample-oriented analytical approach that we are undertaking. The reasons for this will become evident through the derivations we offer in subsequent sections.

### Tip–sample force in intermittent contact of a viscoelastic surface

Now the goal is to develop an analytical equation describing the force in time when the tip has developed a nearly sinusoidal response. From the sample's viewpoint, it is evident that the specimen does not develop a sinusoidal response due to the intermittent-contact character of the interaction. For a closer view of this, let us examine Figure 2. The green solid line represents the position of the viscoelastic sample in time. During some portion of time, tip and sample share the same spatial coordinate. That portion of time starts at time *t*′, when the tip encounters the sample, and ends at time *t*″, when the tip–sample force becomes zero (when the probe leaves the sample). From this observation, we may regard the sample displacement excitation, *h*(*t*), as:

[18]
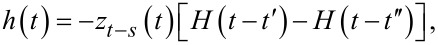


where the negative sign in the tip–sample distance refers to the convention that when tip–sample position is negative, the sample experiences positive deformations (*h*(*t*) > 0). The second term in brackets in Equation 18 makes zero the displacement excitation whenever time is out of the time range of contact, (*t*′,*t*″). As will be shown later, finding the value of *t*″, requires finding the moment when the force in time becomes zero. After that instant, the tip continues its sinusoidal response while the sample experiences recovery (rebound) in a profile related to the strain retardation (creep) function, *J*(*t*) [36]. However, for the first part of the derivation, the value of *t*″ is not available, so we temporarily regard the excitation as:

[16]
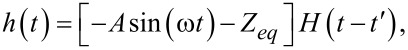


knowing in advance that, at some point, the response calculated through this technique will not be applicable for times greater than *t*″. We will take care of the latter afterwards. For now, the excitation described by Equation 16 can be divided into two individual functions:

[19]
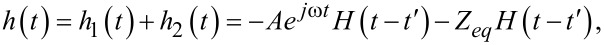


where complex notation is used for mathematical convenience, bearing in mind that (at the end) only the imaginary portion of the response should be kept, after retransformation to the time domain [37–38]. Linear systems allow us to investigate separately the response to each excitation, followed by addition of the responses at the end to find the total response via the superposition principle. Thus, we begin by applying the Laplace transform to the first part of the excitation:

[20]
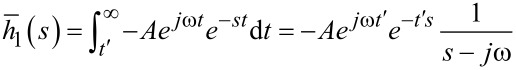


and introducing the result into Equation 1, which gives the transformed force response to the harmonic excitation:

[21]
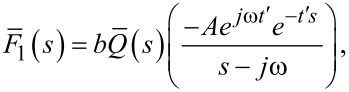


where 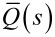 is the relaxance of the material, which is the transfer function connecting the transformed stress and strain,


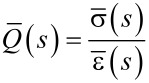


(see Supporting Information File 1 for further details). Here we must develop the full response of the system, because during the small interval of harmonic excitation between *t*′ and *t*″ (see Figure 2), it is not expected that the sample will develop a response only associated to the pole of the driving transform (as it is the case for the techniques studied in the previous section: DMA, contact-resonance AFM, force-modulation AFM). To make the treatment general, we refer to the transfer function (also called relaxance, 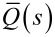) of the model in Figure 1 (see Equation S3 in Supporting Information File 1) and insert it into Equation 21:

[22]
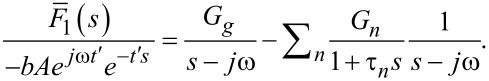


The second term inside the brackets can be decomposed using partial fractions:

[23]
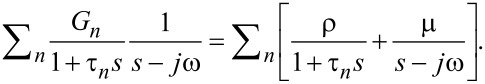


Performing the algebra leads to


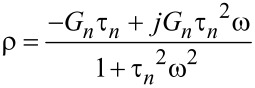


and


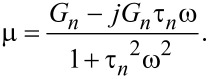


Inserting the above into the right-hand side of Equation 22 yields:

[24]
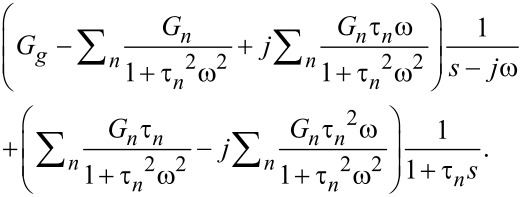


We recognize that the first term of the above equation is the portion of the response associated with the driving transform, and by comparison with Equation S28 and Equation S29 in Supporting Information File 1, we observe that it corresponds to the complex modulus (G*(ω)). The second part of Equation 24 is the part of the response associated with the poles of the material transform, and may be regarded as the transient part of the solution. Implementing the above observations and combining with Equation 22 we obtain:

[25]
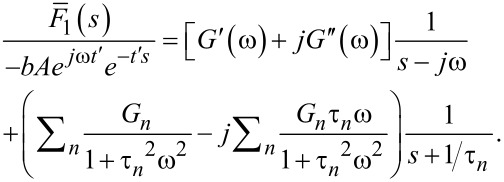


After retransformation of Equation 25, manipulation of the complex algebra, and keeping in mind that only the imaginary portion is meaningful because Im(*e^j^*^ω^*^t^*) was used in the excitation (Equation 19), we obtain:

[26]
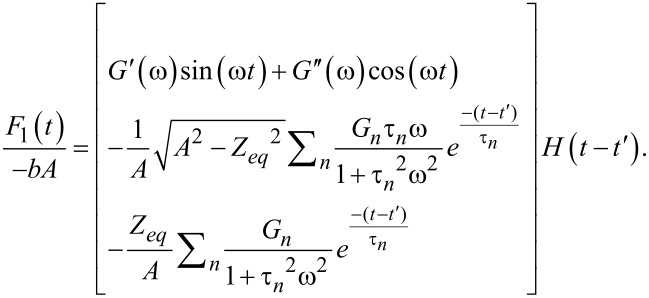


Now, turning our attention to the second part of the excitation in Equation 19 and applying transformation we obtain:

[27]
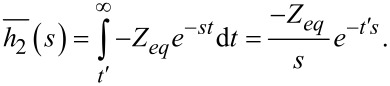


Inserting Equation 27 into Equation 1 and using the relaxance of the Generalized Maxwell model in Figure 1 (see Equation S2 in Supporting Information File 1) leads to:

[28]
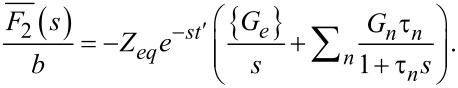


Retransforming Equation 28 gives:

[29]
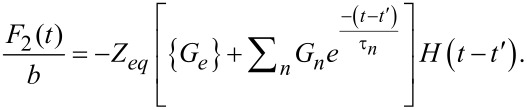


The term in brackets is the stress–relaxation function (stress response to a unit step strain [8]) shifted by the time *t*′, (*G*(*t* − *t*′)). For further details on the stress relaxation of the Generalized Maxwell model see Equation S5 in Supporting Information File 1. Combining Equation 26 with Equation 29, the total solution can be obtained:

[17]
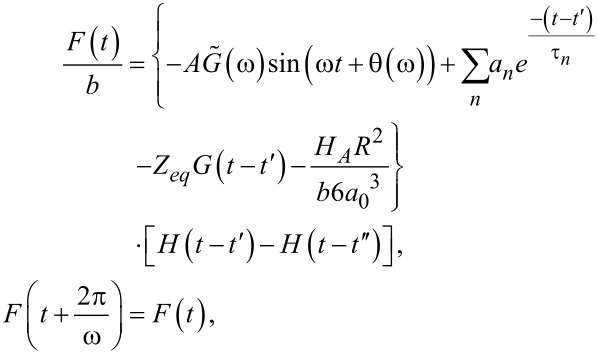


where the relations


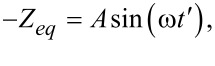



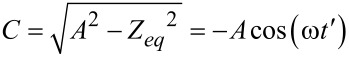


and


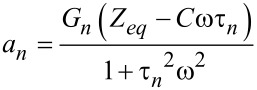


have been introduced. 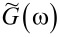 and θ(ω) are the absolute modulus and the loss angle, respectively (for more details see Supporting Information File 1). In Equation 17 it is explicit that the function is periodic with period 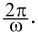 The first term inside the curly brackets refers to the portion of the response associated with the pole of the driving transform (*j*ω, steady-state portion). The second term is the term associated with the poles of the transfer function (−1/τ*_n_*, transient portion) arisen from the harmonic excitation suddenly imposed during the impact. The third term is the relaxation modulus,


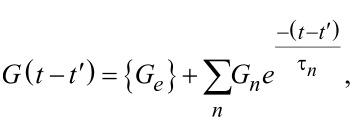


and the fourth term is the adhesive portion of the van der Waals (vdW) interaction, in which *H_A_* is the Hamaker constant, *R* is the radius of the cylindrical punch, and *a*_0_ is the interatomic distance (ca. 0.2 nm) [39]. In Equation 17 we already included *t*″, which is the instant when the force becomes zero (in case of no adhesion) or when


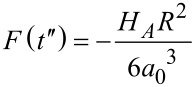


(in the case when vdW adhesion is considered). The above conditions are now used to find *t*″. Due to the complexity of the equation, we calculate *t*″ numerically for the specific cases considered. Note that Equation 17 has been derived (for convenience) assuming that the tip deflection is approximately given by *A*sin(ω*t*). If instead, the more standard expression is chosen (*A*cos(ω*t* − δ), where δ refers to the AFM phase lag), Equation 17 is still fully applicable and only needs to be translated by the appropriate amount in time, specifically: (1/ω)(π/2 – δ).

**Figure 3:**
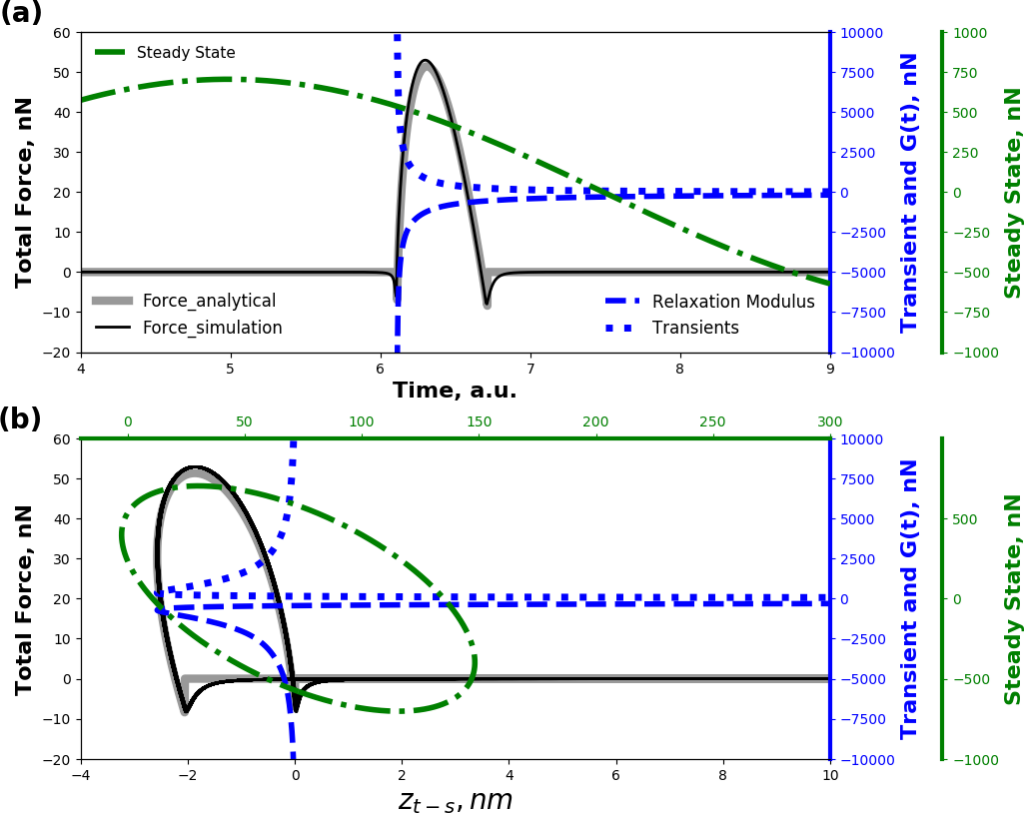
Results for the tip–sample force in tapping-mode AFM, decoupled trough the analytical relation derived (Equation 17). Panel (a) shows the decomposition of the force, plotted vs time, while panel (b) shows the same decomposition but as function of tip–sample position (a common representation in AFM known as force–distance curve). Both panels display the main components of the tip–sample force: i) steady-state harmonic response (green dash-dotted line, first term of Equation 17), ii) transients (blue dotted line, second term of Equation 17), and iii) relaxation modulus (blue dashed line, third term of Equation 17), when a flat-ended rigid indenter interacts in intermittent contact with a viscoelastic sample. The viscoelastic sample is polyisobutylene, represented by a Generalized Maxwell model (see Figure 1) with 26 arms. The parameters of the model were digitalized from the data provided by Brinson and Brinson [25], who fitted the experimental data of Catsiff and Tobolsky [6], and are provided in Table S1 in Supporting Information File 1). The cantilever parameters of the AFM simulation are: *k*_1_ = 10 N/m, *f*_0_ = 100 kHz, *R* = 10 nm, *Q*_1_ = 100, *Q*_2_ = 450, *Q*_3_ = 750. The free oscillation amplitude is *A*_0_ = 100 nm and the reduced tapping amplitude (amplitude setpoint) is *A* = 75 nm.

Figure 3 shows the different contributions to force in time. The force is separated into its components according to Equation 17. The steady-state force is the one associated with the pole of the driving transform, and is represented by the first term in Equation 17 (shown as a green dash-dotted line). This portion of the solution that is oscillatory in time is the dominant component in applications such as DMA, where a harmonic steady-state force and sample displacement are achieved. When analyzing this steady-state component in a plot of force vs tip position (FD curve, Figure 3b), one can observe that the harmonic steady-state force has the shape of an ellipse that is known as a Lissajous ellipse [8].

It is important to clarify here that the loss angle θ(ω) is different from the AFM phase δ(ω), which is the phase lag of the response of the cantilever with respect to its excitation. On the other hand, the loss angle θ(ω) in this context is the phase lead of the (tip–sample) force response with respect to the (sample) local displacement excitation in the steady-state, and its value spans from zero, when a material is completely elastic, to 90°, when the material is completely viscous (see Supporting Information File 1 for further information on θ(ω)).

The portion of the force response associated with the poles of the material transform (second term inside curly brackets in Equation 17) is shown with a blue dotted line in Figure 3. This component of the response is related to the transient that arises when the harmonic excitation is suddenly imposed. In standard harmonic techniques (e.g., DMA, contact-resonance AFM, force-modulation AFM), this portion can be neglected because in those applications it is assumed that a steady-state response has been achieved, and therefore the contribution related to the pole of the driving function dominates (see Equation 5). Unlike steady-state harmonic techniques, in tapping-mode AFM the steady-state response assumption is not appropriate, since at each impact the material gets perturbed from a near original state, and therefore the contributions from the transients cannot be neglected, as it is clear from the results shown in Figure 3. The third term inside the curly brackets in Equation 17 is related to the relaxation modulus (*G*(*t*)), and arises from the position offset between the cantilever average position (equilibrium position) and the initial position of the sample (before tip impact). This term, associated with the relaxation modulus, is plotted in Figure 3 with a blue dashed line. The black thin-solid line in Figure 3 shows the total analytical force as the sum of the three previously described components, and it is evident that none of the components is negligible. Additionally, the results for the numerical simulation are depicted in Figure 3 with a gray thick-solid line, and it is also clear that a close agreement with the analytical solution (Equation 17) exists. This satisfactory agreement has been observed for all cases studied, which illustrates the robustness of the analytical solution.

It is important to note that the analytical solution derived does not consider the attractive portion of the vdW tip–sample interaction (for simplicity), but instead only the portion due to adhesion. The adhesion force is constant during contact,


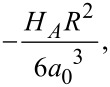


and translates in our analytical equation into a simple offset. Although the adhesion force in Figure 3b does not emerge as a sudden step, but rather builds up gradually according to the vdW attractive interaction, the sudden-step approximation introduces only a small inaccuracy into the energy quantity calculations, as will be shown below.

### Energy quantities for the intermittent contact of an AFM tip with a viscoelastic surface

So far, our analyses have been related to the force in time when a flat-end punch indenter taps harmonically on the viscoelastic surface. Now we turn our attention to the amount of energy that is dissipated during the tapping process. The calculation can be performed by integrating the product of the force (given by Equation 17) with the time derivative of the sample position (Equation 18), as described by Equation 6. Integration yields an expression of energy dissipated per fundamental tapping cycle in terms of the viscoelastic material parameters:

[30]
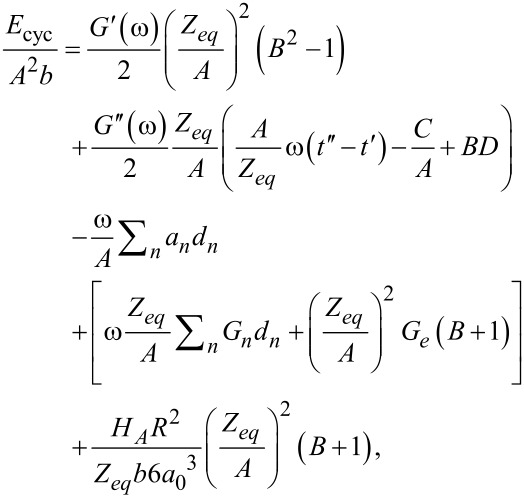


where the relations


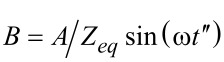


and


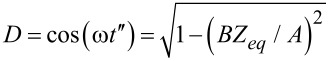


were used. Also,


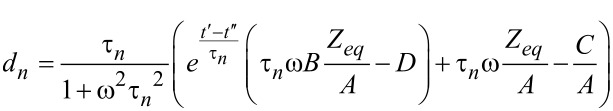


has been substituted for clarity.

The first term in Equation 30 is proportional to the storage modulus *G*′(ω); the second one is proportional to the loss modulus *G″*(ω); the third term is related to the transients (associated with the poles of the transfer function); the fourth term relates to the relaxation modulus (*G*(*t*)); and the fifth term is related to the adhesion component of the vdW interaction (surface energy hysteresis). Interestingly, energy dissipation in this case is not exclusively proportional to the loss modulus as in the case of steady-state harmonic applications (e.g., DMA, see Equation 8). It is evident that substantial complexity is generated in the analytical relations derived when the system does not achieve steady-state (compare Equation 8 to Equation 30). The above is natural because in the tapping case the force is not harmonic (Equation 17), and therefore, its convolution with velocity (Equation 6) to obtain energy dissipation results in a much more complex solution than in the simple DMA case, where both force and displacement oscillate harmonically (Equation 3 and Equation 5).

The calculation of dissipated energy in tapping-mode AFM is well established for high quality factor (high-*Q*) environments [16–17], and has been successfully performed regardless of the source of dissipation in the tip–sample interaction [34,40–41]. However, it is well known that varying the dynamic AFM parameters (e.g., excitation frequency, tapping amplitude) can significantly alter the calculated values of dissipated energy when imaging viscoelastic polymers [35]. This clearly represents a challenge in correlating the values of dissipated energy with meaningful viscoelastic material properties. However, Equation 30 offers a potentially feasible path for extracting material information because it directly relates a quantity that is measurable in real experiments with meaningful viscoelastic properties. Throughout these derivations the shear moduli have been employed, although these quantities can be also expressed in terms of their corresponding tensile moduli (*E*) by using the well-known relation: *E* = 2*G*(1 + ν).

**Figure 4:**
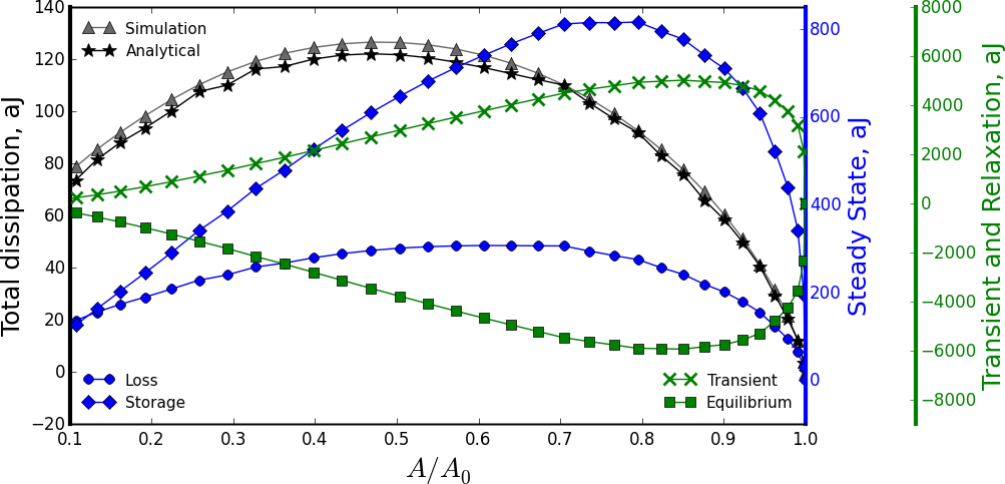
Typical dissipation spectroscopy curve, showing dissipated energy as a function of the ratio between tapping amplitude and free amplitude (*A*/*A*_0_). This plot shows how Equation 30 can decouple the total dissipated energy into meaningful viscoelastic components. The simulation results (see up-triangle symbols) correspond to the case of a flat-end-tip cantilever tapping over a polyisobutylene sample modeled through a Generalized Maxwell model (see Figure 1) with 26 arms (the parameters of the model were digitalized from the data provided by Brinson and Brinson [25], who fitted the experimental data of Catsiff and Tobolsky [6], and are provided in Table S1 in Supporting Information File 1). Star symbols show the analytical calculation based on Equation 30, which closely follows the results obtained from the simulation. The analytical solution specifically gives the amount of dissipated energy that is proportional to the storage modulus (first term in Equation 30, diamond symbols), the amount of dissipated energy that is proportional to the loss modulus (second term in Equation 30, circle symbols), the amount of dissipated energy that is proportional to the transients (third term in Equation 30, cross symbols), and the amount of dissipated energy that is related to the relaxation modulus (fourth term in Equation 30, square symbols). The cantilever parameters of the AFM simulation are the same as the ones given in the caption of Figure 3.

Figure 4 shows the computational results of a dissipation spectroscopy curve (gray triangle symbols), where the cantilever was approached towards the sample by decreasing its equilibrium position (*Z_eq_*, the average tip position with respect to the sample). Each symbol represents a different simulation performed at a different equilibrium position, resulting in a different ratio of tapping amplitude (*A*) to free oscillation amplitude (*A*_0_). As the cantilever approaches the sample, the ratio *A*/*A*_0_ diminishes, and for each *Z_eq_* position the cantilever is allowed to tap for a sufficiently long time to achieve a near steady-state as the amplitude is calculated using the customary in-phase and quadrature terms [35] and the dissipated energy is numerically calculated through the integral


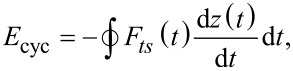


where *F_ts_*(*t*), and *z*(*t*) are the instantaneous tip–sample force and tip deflection, respectively. In the same graph the main components of Equation 30 are plotted, along with the summation of all the components (black star symbols) showing good agreement between the simulations and Equation 30 over the whole range of *A*/*A*_0_.

Besides the main contributors, the adhesive component of the vdW interaction also adds to the total dissipated energy, because the tip–sample trajectory is not symmetric during the contact portion. Instead, the tip remains more time in contact with the sample during the approach than during the retract (see Figure 2). The vdW contribution will thus be referred to as surface energy hysteresis (fifth term on the right-hand side of Equation 30), and is proportional to the mismatch between the surface initial (unperturbed) position and the surface position at which the cantilever leaves the sample (that is, the difference between the two minima in Figure 3b). This contribution was not included in Figure 4, which for clarity was devoted exclusively to the analysis of viscoelastic contributions.

As a final comment on the dissipated energy, we point out that both simulations and analytics assume that dissipation stems exclusively from viscoelastic dissipation and the adhesion force [41–42]. This neglects other sources, such as capillary forces [41], rate dependent adhesion forces, and long-range dissipative interfacial forces [43], among others, which could also play an important quantitative role. This simplification has been made with the purpose of investigating in detail one of the key aspects in materials characterization. As a result, the practical application of the analysis shown here would require a carefully designed experimental setup that minimizes all sources of dissipation that are not related to viscoelasticity. Another important consideration is that, in addition to the viscoelastic material parameters, *Z_eq_* (the average tip position with respect to the sample) is not known in an experiment. This poses a serious problem, since for an actual spectroscopy experiment one would have only one observable, dissipated energy calculated using expressions derived by Cleveland et al., and Tamayo and García [16–17], and at least two unknowns, the material (including all its parameters) and *Z_eq_*. Fortunately, San Paulo and García [18] have shown that, besides energy dissipation, it is possible to obtain another meaningful energy quantity defined as the convolution of the tip–sample interaction force with the tip deflection [44], as described in Equation 9. This is the virial that can be calculated in terms of experimental tapping-mode AFM observables [18], and has been shown to be mathematically independent from the dissipated energy [44–45]. Performing the convolution of force (Equation 17) with sample position, as described in Equatio 9, leads to an expression for the virial for the specific case of a flat-punch probe tapping on a generalized viscoelastic surface:

[31]
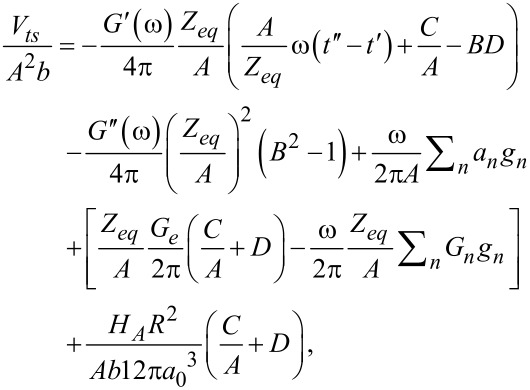


where


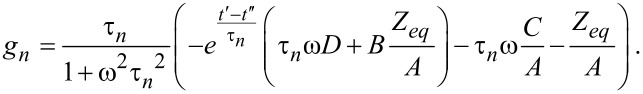


**Figure 5:**
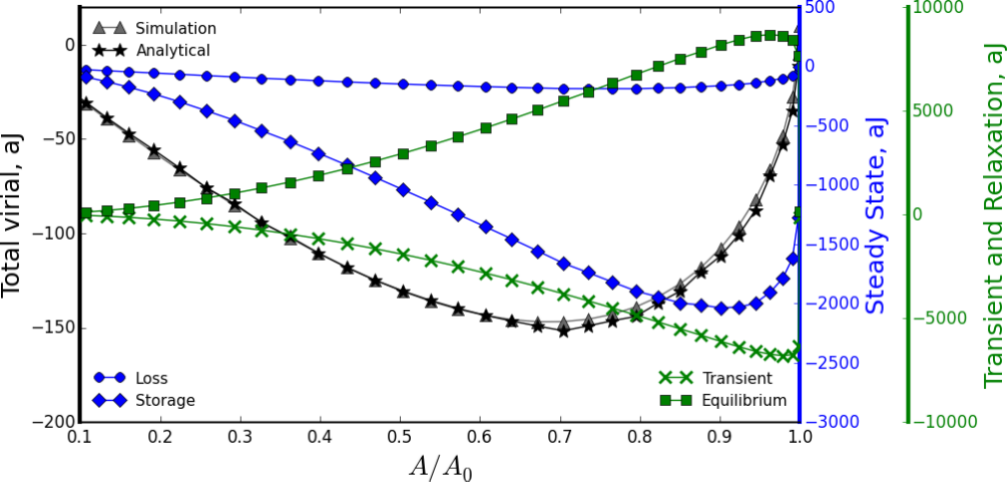
Virial spectroscopy curve, showing the virial as a function of the ratio between tapping amplitude and free amplitude (*A*/*A*_0_). The plot shows how Equation 31 can separate the total virial into meaningful viscoelastic contributions. The simulation results (triangle symbols) correspond to the case of a flat-punch probe tapping on a polyisobutylene sample (this is the same simulation as the one described in the caption of Figure 4). The total virial is decomposed into terms related to *G*′(ω), *G*″(ω), *G*(*t*), and the transients of the harmonic force. The symbol scheme is the same as in Figure 4.

This relationship also enables decomposition of the virial into different terms that are proportional to the viscoelastic material properties. As an example, these different contributions can be visualized in Figure 5 for the case of a flat-punch probe tapping on a polyisobutylene sample (this corresponds to the same numerical simulation used to construct Figure 4). Here it is also evident that the intermittent-contact nature of the interaction forbids the derivation of a simple equation as in the case of DMA (see Equation 11), in which the virial is only proportional to the storage modulus. Here, as for the case of dissipated energy, the virial has contributions that are not only proportional to the storage modulus *G*′(ω), but also to the loss modulus *G*″(ω), the relaxation modulus *G*(*t*) (4th term in Equation 31), and contributions proportional to the transients of the harmonic force (3rd term in Equation 31). All these contributions are plotted in Figure 5, following the same symbol scheme as for the case of dissipated energy in Figure 4. For clarity the contribution from the adhesive force (surface energy) is also omitted.

Although the analytical equations derived here are shown to follow consistently the numerical results for all the cases we have studied, they are still approximations prone to error. The viscoelastic treatment used, based on transformational calculus, carries the intrinsic assumption of zero initial conditions. In other words, we have assumed that each time the cantilever taps on the sample, it finds it at a near rest position with negligible initial conditions (the displacement and force derivatives are assumed to be zero). The above makes the analytics tractable, although it is not formally true, since during sample recovery, the sample follows a trajectory related to the creep compliance function [36,46–47], and it is likely that before full recovery takes place the probe taps on the sample again (see the portion of the sample trajectory after the tip leaves the sample in Figure 2). Since viscoelasticity implies history dependence (fact evidenced by the convolution integral in Equation 2), assuming zero initial conditions neglects previous history and therefore potentially introduces error in the analytical equations. The above however, may have a minor effect in many cases (as it did in all the cases we studied), since the conditions present during surface recovery may be ‘soft’ compared to the time-scale in which the perturbation of the tip acts. In other words, the deformation rates commanded by the tapping process are sufficiently large compared to the deformation rates of sample recovery. Thus, we may refer to these conditions as ‘soft history’. Another source of error of less importance is the contribution to energy dissipation from the excitation of higher modes. For the large-*Q* conditions we studied, we found that the contribution from higher modes is negligible, but errors are expected in high-damping environments. Finally, we have not explored in detail the success of the analytical equations in terms of dynamic parameters (such as free amplitude and amplitude setpoint) but we have found in this exploratory study that typical tapping amplitudes (ca. 100 nm) result in satisfactory agreement with simulation results, regardless the amplitude setpoint used, as shown in Figure 4 and Figure 5, where close consistency was achieved along the whole axis of the reduced ratio between tapping amplitude and free amplitude (*A*/*A*_0_).

A potential practical application of the equations derived here is the following: Dissipated energy and virial may be calculated from the observables of a tapping-mode experiment [16–18] and then equated to the terms in Equation 30 and Equation 31 to obtain material parameters and *Z_eq_*. For a given material (with a given set of parameters) there may be a single *Z_eq_* value that satisfies both quantities (dissipated energy and virial), and therefore these two quantities may work together complementary to give the unknown *Z_eq_*. Because the material properties represent more than one unknown, an unambiguous approximation of the material parameters would require having more than one data point, which in fact is not a limitation within amplitude and phase spectroscopy experiments (as illustrated in Figure 4). The design of a numerical algorithm capable of extracting material properties is beyond the scope of this study, although it seems to be a feasible task that could eventually exploit the relations we have derived. We expect such algorithm to be more successful in material property extraction when a higher number of data points is available, although this could potentially undermine a key advantage of tapping-mode AFM, which is its high scanning speed. At the same time, knowing that the observation of viscoelasticity demands agreement between the material timescale (e.g., relaxation time) and the experimental timescale (ca. 1/ω), we expect that in tapping mode AFM the observables are mainly governed by the elements of the model whose relaxation times are nearest to the inverse of the tapping frequency. The above has important implications with regards to the simplification of the model to be considered in each particular case (i.e., the number of arms retained in the Generalized Maxwell model), which determines the number of unknowns to be solved for, and consequently, the number of data points to be acquired for a successful fitting.

The analytical relations derived here portray the complexities of the deformation of viscoelastic materials in tapping mode AFM, and they may seem rather daunting to the general user. Therefore, we anticipate that a useful research outlook would focus on seeking engineering approximations that could simplify these expressions, along with specific experimental conditions where those simplified expressions would be appropriate. We believe that our rigorous analytical expressions provide a solid ground for the exploration of such simplifications.

## Conclusion

We have studied thoroughly the physics of a flat-punch AFM probe tapping on a generalized linear viscoelastic surface containing an arbitrary number of characteristic times. We have derived analytical expressions for force in time and for two energy quantities frequently used in tapping-mode AFM, namely the average dissipated energy and the virial, in terms of meaningful viscoelastic material properties. We have derived the expressions from the material point of view, using rigorous linear viscoelasticity theory in a general manner, such that the treatment is applicable for real materials. This material-focused rheological approach (defining an input displacement of the surface and deriving the corresponding output force) is a complementary approach to the linear dynamics strategy usually followed by the AFM community, which focuses on the cantilever motion. We anticipate that combining these two approaches can lead to a practical use of the expressions derived here. Our expressions shed light into the complexity of the tip–sample force term, and the specific contributions of viscoelastic properties (*G*′(ω), *G*″(ω), *G*(*t*)), which can be counterintuitive. A thorough comparison of energy quantities for tapping-mode AFM with those of steady-state techniques (e.g., DMA) allows us to understand the important differences among these methods, as well as the reasons behind the challenges that emerge when attempting to extract meaningful material properties in tapping-mode AFM, where oversimplified assumptions are frequently used, which are not appropriate for viscoelastic materials. Flat-punch indentation has been chosen for two main reasons: i) to ensure full applicability of the correspondence viscoelastic principle, and ii) to keep the analytics workable. Although this only represents a portion of the general problem of indentation of viscoelastic materials by arbitrary profiled AFM indenters, it is a step forward in terms of understanding the complexities of the technique in the context of viscoelastic materials, as well as the physical quantities governing the observables.

## Methods: Numerical Simulations

To model the dynamics of the cantilever, a system of three ordinary differential equations is used, in which each equation corresponds to one eigenmode of the cantilever (assuming the dynamics are mainly contained in the first three eigenmodes) [32]:

[32]
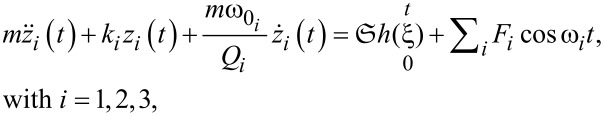


where *m* is the effective mass of the cantilever, *z_i_* is the *i*-th eigenmode displacement, *k_i_* is the *i*-th eigenmode force constant, 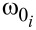 is the *i*-th eigenmode resonance frequency, *F_i_*, ω*_i_*, and *Q_i_* are the force amplitude, excitation frequency, and quality factor of the *i*-th eigenmode, respectively. The tip deflection is *z*(*t*) = Σ*_i _z_i_*(*t*), and the tip–sample distance is *z_t_*_−_*_s_*(*t*) = *z*(*t*) + *Z_eq_*, where *Z_eq_* is the average tip position with respect to the sample.

The notation employed to represent the tip–sample force term,


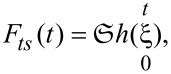


in Equation 32 emphasizes the nature of the viscoelastic material modeled. According to it, the tip–sample force is a functional of the sample deformation. In other words, the force at the current time *t, F_ts_*(*t*)*,* depends on the history of the surface deformation at all previous times ξ, from ξ = 0 to ξ = *t* [8,13]. This force term is included in the simulation by implementing Cheng’s solution in operator equation form for the flat-punch case [28]:

[33]
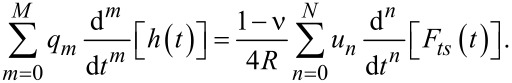


The constants *q_m_* and *u_n_* can be found by algebraic manipulation of the relaxance of the material (Equation S2 or Equation S3 in Supporting Information File 1), which is a ratio of polynomials 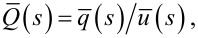 where the numerator and denominator are defined as:

[34]
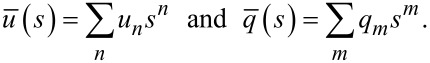


Therefore, the coefficients *q_m_* and *u_n_* can be found by grouping the coefficients of the relaxance according to their power in the complex variable ‘*s*’ (for additional details refer to [8,13]). After finding the coefficients of the differential equation in Equation 33, its numerical calculation is performed as follows: when the tip is in contact with the sample, then *z_t_*_−_*_s_*(*t*) = −*h*(*t*), and the time derivatives of sample deformation can be calculated up to the *M*-th order. Afterwards, the *N*-th order derivative is calculated on the force


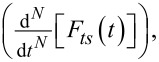


followed by the calculation of the lower order derivatives, down to the zero-th order, which corresponds to the value of *F_ts_*(*t*)*.* Additionally, the vdW interaction has been included as


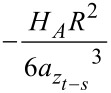


during the noncontact portion of the interaction. This expression was derived through pairwise addition using the non-retarded Hamaker summation method [39]. Here the total attractive van der Waals potential (*E*_vdW_) was calculated for the specific case of a flat-end cylindrical punch interacting with a flat semi-infinite half-space, and subsequently the interaction force was obtained through differentiation:


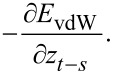


For the adhesion portion during contact, a constant adhesion force was added as


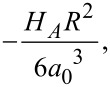


as already explained.

The calculation of the tapping amplitude (*A*) for the construction of the dissipative and virial spectroscopy curves (Figure 4) was performed by extracting the Fourier components of the tip deflection (*z*(*t*)) that are related to the driving frequency, using the customary in-phase and quadrature terms [21]. The numerical calculations of the dissipated energy were performed through the integral 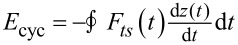 [45], while the numerical calculations of the virial were performed using the relation 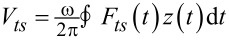 [32]. For additional details of the simulations and analytical solutions refer to the open access code provided in [48].

## Supporting Information

File 1 This file contains relevant information related to the theory of linear viscoelasticity that may help the reader follow the analytical derivations. It also contains information related to the viscoelastic material that was used in the numerical simulations.

## References

[1] Silberstein M N, Boyce M C (2010). J Power Sources.

[2] Dittmer J J, Lazzaroni R, Leclère P, Moretti P, Granström M, Petritsch K, Marseglia E A, Friend R H, Bredas J L, Rost H (2000). Sol Energy Mater Sol Cells.

[3] Arrechea S, Aljarilla A, de la Cruz P, Palomares E, Sharma G D, Langa F (2016). Nanoscale.

[4] Lekka M, Laidler P (2009). Nat Nanotechnol.

[5] Noh H, Diaz A J, Solares S D (2017). Beilstein J Nanotechnol.

[6] Catsiff E, Tobolsky A V (1955). J Colloid Sci.

[7] Schapery R (1962). Proc. Fourth US Nat. Congress of Appl. Mech..

[8] Tschoegl N W (2012). The Phenomenological Theory of Linear Viscoelastic Behavior: an Introduction.

[9] Yablon D G, Grabowski J, Chakraborty I (2014). Meas Sci Technol.

[10] Yuya P A, Hurley D C, Turner J A (2008). J Appl Phys.

[11] Radmacher M, Tillmann R W, Gaub H E (1993). Biophys J.

[12] Cartagena A, Raman A (2014). Biophys J.

[13] López-Guerra E A, Eslami B, Solares S D (2017). J Polym Sci, Part B: Polym Phys.

[14] Koppensteiner J, Schranz W, Puica M R (2008). Phys Rev B.

[15] Young S K, Mauritz K A (2001). J Polym Sci, Part B: Polym Phys.

[16] Cleveland J P, Anczykowski B, Schmid A E, Elings V B (1998). Appl Phys Lett.

[17] Tamayo J, Garcıa R (1998). Appl Phys Lett.

[18] San Paulo Á, García R (2001). Phys Rev B.

[19] Solares S D (2015). Beilstein J Nanotechnol.

[20] Solares S D (2016). Beilstein J Nanotechnol.

[21] López-Guerra E A, Solares S D (2014). Beilstein J Nanotechnol.

[22] Williams J C, Solares S D (2012). Proceedings of the Asme International Design Engineering Technical Conferences and Computers and Information in Engineering Conference, Vol. 7.

[23] Alfrey T (1944). Q Appl Math.

[24] Lee E H (1955). Q Appl Math.

[25] Brinson H F, Brinson L C (2008). Polymer Engineering Science and Viscoelasticity.

[26] Peng Y, Zhou D (2012). J Comput Modell.

[27] Zhu H-H, Liu L-C, Pei H-F, Shi B (2012). Geomech Geoeng.

[28] Cheng L, Xia X, Yu W, Scriven L E, Gerberich W W (2000). J Polym Sci, Part B: Polym Phys.

[29] Sneddon I N (1965). Int J Eng Sci.

[30] Ferry J D (1980). Viscoelastic Properties of Polymers.

[31] Stan G, Solares S D, Pittenger B, Erina N, Su C (2014). Nanoscale.

[32] Lozano J R, Garcia R (2008). Phys Rev Lett.

[33] Roylance D (2016). "Engineering viscoelasticity". Massachusetts Institute of Technology, 2001.

[34] Eslami B, López-Guerra E A, Raftari M, Solares S D (2016). J Appl Phys.

[35] Diaz A J, Eslami B, López-Guerra E A, Solares S D (2014). J Appl Phys.

[36] Argatov I, Mishuris G (2011). Mech Res Commun.

[37] Meirovitch L (2010). Fundamentals of Vibrations.

[38] Gardner M F, Barnes J L (1956). Transients in Linear Systems Studied by the Laplace Transformation v.1.

[39] Israelachvili J N (2015). Intermolecular and Surface Forces.

[40] Martinez N F, García R (2006). Nanotechnology.

[41] Zitzler L, Herminghaus S, Mugele F (2002). Phys Rev B.

[42] Derjaguin B V, Muller V M, Toporov Yu P (1975). J Colloid Interface Sci.

[43] Garcia R, Gómez C J, Martinez N F, Patil S, Dietz C, Magerle R (2006). Phys Rev Lett.

[44] García R (2011). Amplitude Modulation Atomic Force Microscopy.

[45] Lozano J R, Garcia R (2009). Phys Rev B.

[46] Argatov I (2012). Acta Mech.

[47] Argatov I I, Popov V L (2016). Z Angew Math Mech.

[48] (2017). flat_punch.

