# Supplementary material for: Material property analytical relations for the case of an AFM probe tapping a viscoelastic surface containing multiple characteristic times
Source: Beilstein J Nanotechnol. 2017 Oct 26;8:2230–44. doi: 10.3762/bjnano.8.223 (PMC5669240; doi:10.3762/bjnano.8.223)
Supplement: File 1 — This file contains relevant information related to the theory of linear viscoelasticity that may help the reader follow the analytical derivations. It also contains information related to the viscoelastic material that was used in the numerical simulations. [file Beilstein_J_Nanotechnol-08-2230-s001.pdf]

Supporting Information

for

Material property analytical relations for the case of an AFM probe tapping a viscoelastic surface containing multiple characteristic times

Enrique A. López-Guerra and Santiago D. Solares[*,§]

Address: Department of Mechanical and Aerospace Engineering, The George Washington University, Washington, DC 20052, USA

Email: Santiago D. Solares - ssolares@gwu.edu

*Corresponding author

[§]Phone: +1 (202) 994-0372

1. Transfer Functions in Linear Viscoelasticity

Typically, linear viscoelasticity is treated through spring-dashpot models, which are able to reproduce the intricate physical relationships between stress and strain [1-3]. For a general treatment where a surface has multiple relaxation times, as is the case for many real materials, the Generalized Maxwell (also called Wiechert) Model may be used (see Figure 1 in the main manuscript). For this case, the transfer function relating the Laplace transformed strain ($\bar{\varepsilon}(s)$) and the Laplace transformed stress ($\bar{\sigma}(s)$), when strain is regarded as the input, is the *relaxance* $\bar{Q}(s)$:

$$\bar{Q}(s) = \frac{\bar{\sigma}(s)}{\bar{\varepsilon}(s)} \tag{S1}$$

The relaxance for spring-dashpot models can be obtained directly in the transform plane using techniques analogous to those used for determining mesh equations in electric circuit theory [1,4]. Briefly, relaxances are added in parallel and retardances (inverses of relaxances) are added in series. The relaxances of the springs and dashpots (in the mechanical model diagram of Figure 1) are G_n and $\eta_n s$, respectively (G_n is the modulus of the nth spring, and η_n is the viscosity of the nth dashpot). The retardances of elements in series (in a Maxwell arm) may be added to get the retardance of the nth arm: $\frac{1}{G_n} + \frac{1}{\eta_n s}$, and its inverse will be the relaxance of the nth arm: $\frac{G_n \eta_n s}{G_n + \eta_n s}$. Summation over all the arms (in parallel) and making the substitution: $\tau_n = \eta_n / G_n$ (relaxation time of the nth arm), leads to the total relaxance of the model:

$$\bar{Q}(s) = \{G_e\} + \sum_n \frac{G_n \tau_n s}{1 + \tau_n s} \tag{S2}$$

which can also be expressed as:

$$\bar{Q}(s) = G_g - \sum_n \frac{G_n}{1 + \tau_n s} \tag{S3}$$

where $G_g = \{G_e\} + \sum_n G_n$, is the glassy modulus, also called instantaneous modulus, which describes the response of the material at short loading times. G_e is the equilibrium modulus [5], also called rubbery modulus, which describes the material behavior at long time-scales. G_e appears in curly brackets because it may or may not exist. It is non-zero if the material is arrheodictic, meaning that it cannot sustain steady-state flow, or it may be zero if the material displays steady-state flow [1].

The relaxance $\bar{Q}(s)$ may be related to the so-called *relaxation modulus G(t),* which is the material's stress response to a unit step strain *H(t)* (the unit step function). The transform of the strain input, $\varepsilon(t) = H(t)$, is $\bar{\varepsilon}(s) = 1/s$. Inserting this input into Equation (S1) yields:

$$\bar{\sigma}(s) = \frac{\bar{Q}(s)}{s} = \bar{G}(s) \qquad \text{(S4)}$$

Applying the above to Equation (S2) leads to the (transformed) relaxation modulus of the model in Figure 1, which after retransformation gives:

$$G(t) = \{G_e\} + \sum_n G_n e^{\frac{-t}{\tau_n}} \qquad \text{(S5)}$$

More specifically, the above expression corresponds to the *shear* relaxation modulus, which describes the response of the *shear* stress $\sigma_{xy}(t)$ to a unit step engineering *shear* strain $\varepsilon_{xy}(t)$. This notation with the subscripts '*xy*' highlights the tensorial nature of stress and strain, which was disregarded earlier for simplicity (the linear viscoelastic treatment can be extended to the three-dimensional case when needed).

In an analogous way, when the stress is regarded as the input, the transfer function ($\bar{U}(s)$) relating the transformed stress to the transformed strain response is:

$$\bar{U}(s) = \frac{\bar{\varepsilon}(s)}{\bar{\sigma}(s)} \qquad \text{(S6)}$$

Where $\bar{U}(s)$ is also called the retardance, and is the reciprocal of the relaxance ($\bar{U}(s) = 1/\bar{Q}(s)$).

The retardance can also be related to the more extensively used compliance function. The compliance, $J(t)$, also called creep compliance or strain retardation, is the strain response in time ($\varepsilon(t)$) to a unit step stress ($\sigma(t) = H(t)$). The transformed input stress is $\bar{\sigma}(s) = 1/s$, which upon insertion into Equation (S6) yields the transformed compliance ($\bar{J}(s)$):

$$\bar{\varepsilon}(s) = \frac{\bar{U}(s)}{s} = \bar{J}(s) \tag{S7}$$

2. Harmonic Excitation of Viscoelastic Materials: Derivation of Equation (4) in the Main Manuscript

As stated in the main manuscript, the input for experimental cases where the tip is always in contact with the sample may be regarded as:

$$F(t) = F_s H(t) + F_0 Im\left[e^{j\omega t}\right] \tag{S8}$$

where $j = \sqrt{-1}$, and *Im*[ ] indicates that only the imaginary component of the term in brackets is taken into account. Equation (S8) is the same as Equation (3) in the main manuscript, but for mathematical convenience in the derivations, we have used $Im\left[e^{j\omega t}\right]$ instead of its equivalent, $\sin(\omega t)$. Therefore, after retransformation of the response to the time domain, only the imaginary portion will be meaningful.

The transformed force input ($\bar{F}(s)$) is:

$$\bar{F}(s) = \frac{F_s}{s} + F_0\left(\frac{1}{s - j\omega}\right) \tag{S9}$$

where F_s is the static force setpoint, F_0 is the amplitude of the harmonic excitation (tip-sample force), and ω is the driving and response frequency.

Cheng et.al. [6] have applied the *viscoelastic correspondence principle* to extend Sneddon's elastic solution [7] of a flat-end indenter penetrating an elastic half-space to its viscoelastic counterpart. They have shown that for the case of a time-independent Poisson's ratio (ν), the relation between the transformed force ($\bar{F}(s)$) and displacement ($\bar{h}(s)$) can be

written as $\bar{F}(s) = \frac{4R}{1-\nu}\bar{Q}(s)\bar{h}(s)$, which may also be conveniently expressed in terms of the material's retardance $\bar{U}(s)$:

$$\bar{h}(s) = \frac{1}{b}\bar{U}(s)\bar{F}(s) \tag{S10}$$

where the substitution $b = \frac{4R}{1-\nu}$, has been used. The constant b will be regarded as a cell constant (or apparatus constant) [1]. The excitation expression in Equation (S9) can be divided into two parts. Each part can be handled independently and both parts can be added up at the end, thanks to system's time linearity. Focusing on the first part, $\bar{F}_1(s) = \frac{F_s}{s}$, and inserting it into Equation (S10) with the aid of Equation (S7) leads to the displacement response: $\bar{h}_1(s) = \frac{F_s}{b}\bar{J}(s)$, which after retransformation gives:

$$h_1(t) = \frac{F_s}{b}J(t) \tag{S11}$$

Thus, the static force setpoint is associated with a displacement response that is proportional to the material's creep compliance ($J(t)$).

Now, we turn our attention to the second portion of the force excitation in Equation (S9), $\bar{F}_2(s) = F_0\left(\frac{1}{s-j\omega}\right)$, and insert it into Equation (S10) to obtain:

$$\bar{h}_2(s) = \frac{F_0}{b}\frac{\bar{U}(s)}{s-j\omega} \tag{S12}$$

Being the retardance a ratio of polynomials in the complex variable 's', it can be expressed as: $\bar{U}(s) = \bar{u}(s)/\bar{q}(s)$. Substituting the above into Equation (S12) and rearranging leads to:

$$\frac{b}{F_0}\bar{h}_2(s) = \frac{\bar{u}(s)/\bar{q}(s)}{s-j\omega} \tag{S13}$$

The term in brackets of the right hand side of the above equation can be decomposed using partial fractions:

$$\frac{\beta}{s-j\omega} + \frac{\bar{\gamma}(s)}{\bar{q}(s)} = \frac{\beta\bar{q}(s) + \bar{\gamma}(s)(s-j\omega)}{(s-j\omega)\bar{q}(s)} \tag{S14}$$

where β is the portion of the response associated with the pole of the driving transform, and $\bar{\gamma}(s)$ is the portion of the response associated with the poles of the material's relaxance. From Equation (S14) we note that:

$$\beta\bar{q}(s) + \bar{\gamma}(s)(s - j\omega) = \bar{u}(s) \qquad \text{(S15)}$$

In the steady state, when $s = j\omega$, from Equation (S15) we obtain the value of β:

$$\beta = \overline{U}(s)\big|_{s=j\omega} = J^{*}(\omega) \qquad \text{(S16)}$$

The above shows that the retardance in the steady state (when $s = j\omega$; the pole of the driving function) becomes what is commonly known as the *complex compliance*, which describes the displacement response of the material in the steady state when a harmonic force excitation is applied.

Substituting Equation (S16) into Equation (S12), leads to the portion associated with the steady state: $\bar{h}_2{}^{ss}(s) = \frac{F_0}{b}\frac{J^{*}(\omega)}{s-j\omega}$, which after retransformation becomes:

$$h_2{}^{ss}(t) = \frac{F_0}{b}\,\mathrm{Im}\big[J^{*}(\omega)e^{j\omega t}\big] \qquad \text{(S17)}$$

where, as mentioned earlier, we only keep the imaginary component, since we used $Im\big[e^{j\omega t}\big]$ instead of $\sin(\omega t)$ when we defined the input in Equation (S8).

As with any complex quantity, the complex compliance can be expressed in polar coordinates:

$$J^{*}(\omega) = \tilde{J}(\omega)e^{-j\theta(\omega)} \qquad \text{(S18)}$$

where $\tilde{J}(\omega)$ is the absolute compliance, and $\theta(\omega)$ –the loss angle– is the phase lag (or lead) of the response of a viscoelastic material to a harmonic excitation in the steady state. By convention, it is defined that force always leads the displacement, no matter which one is regarded as excitation or response. This explains the negative sign in the exponential in Equation (S18), which emphasizes that the displacement is lagging behind the force by $\theta(\omega)$. The value of $\theta(\omega)$ spans from zero, when a material is completely elastic, to 90 °, when the

material is completely viscous. Furthermore, it is possible to decompose the complex compliance into its real and imaginary parts in rectangular coordinates:

$$J^*(\omega) = J'(\omega) - jJ''(\omega) \tag{S19}$$

where $J'(\omega)$ refers to the storage compliance, and $J''(\omega)$ to the loss compliance. Inserting Equation (S19) into Equation (S17) yields:

$$h_2{}^{ss}(t) = \frac{F_0}{b}[J'(\omega)\sin\omega t - J''(\omega)\cos\omega t] \tag{S20}$$

Recalling that the harmonic force excitation is: $F_2(t) = F_0 \sin\omega t$, Equation (S20) shows that the steady state response contains one portion (proportional to $J'(\omega)$) that is in-phase with the excitation and is therefore regarded as the elastic component. The other portion (proportional to $J''(\omega)$) is the viscous component. Combining Equation (S20) with Equation (S11) to get the total response for the total excitation force (Equation (S8)), we obtain:

$$h(t) = \frac{F_s}{b}J(t) + \frac{F_0}{b}[J'(\omega)\sin(\omega t) - J''(\omega)\cos(\omega t)] \tag{S21}$$

which is the same as Equation (4) in the main manuscript.

In a completely analogous way, we may derive the complex modulus ($G^*(\omega)$), which describes the stress (or force) response to a harmonic strain (or displacement) excitation. Briefly, for a harmonic strain excitation ($\varepsilon_0 e^{j\omega t}$), the *steady-state* stress response of a viscoelastic material is given by $\sigma(\omega) = G^*(\omega)\varepsilon(\omega)$. $G^*(\omega)$ is the portion of the response associated with the pole of the driving transform ($\varepsilon_0/(s - j\omega)$), i.e., the steady state, and therefore:

$$G^*(\omega) = \overline{Q}(s)\big|_{s=j\omega} \tag{S22}$$

$G^*(\omega)$ can also be expressed in polar coordinates:

$$G^*(\omega) = \tilde{G}(\omega)e^{j\theta(\omega)} \tag{S23}$$

where $\tilde{G}(\omega)$ is the absolute modulus, and $\theta(\omega)$ is again the loss angle (previously defined). Here, the positive sign in the exponential obeys the convention that the force leads the displacement. Also, $G^*(\omega)$ may be expressed in Cartesian coordinates:

$$G^*(\omega) = G'(\omega) + jG''(\omega) \tag{S24}$$

By relating Cartesian and polar coordinates, and with the aid of Euler's identity, it is easily ascertained that:

$$\tilde{G}(\omega) = \sqrt{\left(G'(\omega)\right)^2 + \left(G''(\omega)\right)^2} \tag{S25}$$

It may also be confirmed that:

$$G'(\omega) = \tilde{G}(\omega)\cos\theta(\omega) \qquad G''(\omega) = \tilde{G}(\omega)\sin\theta(\omega) \tag{S26}$$

Thus,

$$\tan\theta(\omega) = \frac{G''(\omega)}{G'(\omega)} = \frac{J''(\omega)}{J'(\omega)} \tag{S27}$$

where the value of $\theta(\omega)$ remains always positive due to the chosen convention of displacement always lagging behind the force.

Equations (S22) and (S3) can be combined to obtain the complex modulus of the Generalized Maxwell Model shown in Figure 1 of the main manuscript. Afterwards, decomposition into real and imaginary components leads to:

$$G'(\omega) = G_g - \sum_n \frac{G_n}{1 + \omega^2 {\tau_n}^2} \tag{S28}$$

for the storage modulus, and

$$G''(\omega) = \sum_n \frac{G_n \omega \tau_n}{1 + \omega^2 {\tau_n}^2} \tag{S29}$$

for the loss modulus.

3. Viscoelastic Data Used in the Simulations

For the simulations described in Figures 3 to 5 of the main manuscript, the viscoelastic sample was represented with a Generalized Maxwell Model (see Figure 1 in the main manuscript) with 26 Maxwell arms. The corresponding parameters were digitalized from the data provided by Brinson and Brinson (page 249, Fig. 7.19 in reference [2]), who obtained the values by fitting the experimental data of Catsiff and Tobolsky [8]. The digitalized values are summarized in table S1.

Table S1 *Generalized Maxwell Parameters for Poly-isobutylene given by Brinson and Brinson [2].*

Element number	Relaxation time τ (s)	Modulus (Pa)
**1**	1.166E-09	4.132E+08
**2**	4.852E-09	8.227E+08
**3**	2.250E-08	6.315E+08
**4**	9.652E-08	3.607E+08
**5**	3.832E-07	1.533E+08
**6**	1.671E-06	4.522E+07
**7**	7.196E-06	2.230E+07
**8**	2.888E-05	6.101E+06
**9**	1.479E-04	2.606E+06
**10**	5.871E-04	1.108E+06
**11**	2.361E-03	2.816E+05
**12**	9.355E-03	1.288E+05
**13**	4.028E-02	6.354E+04
**14**	1.798E-01	7.212E+03
**15**	8.160E-01	1.336E+04
**16**	3.293E+00	9.276E+04
**17**	1.303E+01	4.567E+04
**18**	5.847E+01	1.315E+05
**19**	2.967E+02	8.110E+04
**20**	1.046E+03	1.390E+05
**21**	5.278E+03	1.068E+05

**22**	2.635E+04	1.276E+05
**23**	8.797E+04	6.263E+04
**24**	4.124E+05	3.094E+04
**25**	1.831E+06	1.384E-01
**26**	7.757E+06	1.322E-01

References

[1] N. W. Tschoegl, *The Phenomenological Theory of Linear Viscoelastic Behavior: an Introduction* (Springer Science & Business Media, 2012).

[2] H. F. Brinson; L. C. Brinson, *Polymer Engineering Science and Viscoelasticity* (Springer, 2008).

[3] López-Guerra, E.A.; Solares, S.D. *Beilstein Journal of Nanotechnology* **2014,** *5,* 2149.

[4] Gardner, M.F.; Barnes, J.L. *Transients in Linear Systems Studied by the Laplace Transformation* (J. Wiley & Sons, inc., 1956).

[5] Ferry, J.D. *Viscoelastic Properties of Polymers* (John Wiley & Sons, 1980).

[6] Cheng, L.; Xia, X.; Yu, W.; Scriven, L.; Gerberich, W. *Journal of Polymer Science Part B: Polymer Physics* **2000,** *38,* 10.

[7] Sneddon, I.N. *International Journal of Engineering Science* **1965,** *3,* 47.

[8] Catsiff, E.; Tobolsky, A. *Journal of Colloid Science* **1955,** *10,* 375.
